# Evaluation of Ammonium Nitrate(V) Morphology and Porosity Obtained by SEM and Tomography Imaging

**DOI:** 10.3390/ma17133156

**Published:** 2024-06-27

**Authors:** Andrzej Biessikirski, Grzegorz Piotr Kaczmarczyk, Łukasz Kuterasiński, Malwina Kolano, Mateusz Pytlik

**Affiliations:** 1 Faculty of Civil Engineering and Resource Management, AGH University of Krakow, 30-059 Krakow, Poland; grzegorz.kaczmarczyk@agh.edu.pl (G.P.K.); mkolano@agh.edu.pl (M.K.); 2 Jerzy Haber Institute of Catalysis and Surface Chemistry, Polish Academy of Sciences, 30-239 Krakow, Poland; lukasz.kuterasinski@ikifp.edu.pl; 3 Conformity Assessment Body, Central Mining Institute—National Research Institute, 40-166 Katowice, Poland; mpytlik@gig.eu

**Keywords:** ammonium nitrate(V), prill, fertilizer, XRD, tomography, SEM

## Abstract

This paper presents an evaluation of the morphology of fertilizer-grade and prill-grade ammonium nitrate(V). All samples were analyzed using X-ray powder diffraction (XRD), scanning electron microscopy (SEM) and tomography techniques. The XRD results revealed that despite various provenances, all samples exhibited similar P_mmm_ symmetry and diffraction patterns. SEM images indicated that prill ammonium nitrate(V) showed a more complex external and internal crystal structure than fertilizer-grade counterparts. Furthermore, tomography analysis revealed that each prill ammonium nitrate(V) sample demonstrated distinct porosity characteristics, including varying pore sizes and distribution patterns. Both methods confirmed that fertilizer-grade ammonium nitrate(V) in the cross-section had a pumice structure, and porous prill ammonium nitrate(V) had a rather complex structure, with a central cavity observed only in the case of Sample 4. The appearance of a central cavity can be explained by the different conditions or manufacturing processes of porous prill ammonium nitrate(V). Moreover, the fertilizer-type ammonium nitrate(V) exhibited the lowest surface-to-volume ratio of ca. 21% compared to the porous-type ammonium nitrate(V). This, together with the lowest surface area of ca. 116 mm^2^, confirmed the lowest absorption capacity of the fertilizer-grade ammonium nitrate(V) disclosed by the ammonium nitrate(V) producer.

## 1. Introduction

Ammonium nitrate(V) (AN) is primarily recognized as a white crystalline, odorless salt. It can exist in various physical forms, such as prills, granules, pellets or flakes. Under normal pressure, ammonium nitrate(V) crystalizes in five distinct phases, designated as I (ε-regular), II (δ-trigonal), III (γ-rhombic), IV (β-rhombic) and V (α-tetragonal) [1,2]. Each phase occurs in specific temperature ranges. Leonard et al. stated that, in 2017, the global AN production reached an estimated 15.3 million tons, with market projections expecting it to reach USD 6.18 billion by 2025 [3], underscoring its significance to the global economy.

AN finds extensive application in agriculture due to its high nitrogen content (approximately 35%) and excellent water solubility, facilitating deep penetration into the root zone. The effectiveness of soil-applied fertilizers is primarily determined by their capacity to sustain adequate nutrient concentrations within the plant root zone over a desired period [3,4]. Nitrogen migration is possible, especially under moist conditions; however, the concentrations of active ingredients diminish rapidly due to various factors, including chemical, photochemical and biological degradation, volatilization, leaching, adsorption and soil immobilization [5]. To overcome this problem, fertilized ammonium nitrate(V) is usually coated with an inert material [6]. The coating can be visible under SEM. 

Fertilizer-grade AN typically occurs as dense granules with low retention properties. On the other hand, porous prill ammonium nitrate(V) serves diverse industrial needs, such as in open-pit mining, necessitating high absorption capabilities, often associated with lower density and higher retention characteristics. Retention is influenced by porosity and governs the application of porous-type AN. In the case of non-ideal highly energetic materials, the porosity and sample morphology influence caking, rheology, attrition and friability [7], material performance [8,9,10,11] and sensitivity [12]. However, different types of ammonium nitrate prills can vary significantly [3]. This results in different sample morphologies and porosities, including samples of similar provenances [13], which leads to various absorption indices. Fertilizer-grade AN typically ranges up to approximately 8%, while prill AN may exhibit absorption in the 8–16% range.

AN’s physicochemical properties vary based on its provenance, which is influenced by the production methods [14,15,16,17,18]. Lotspeich and Petr, Biessikirski et al., as well as Biessikirski and Kuterasiński studied the morphology of AN mini-prills, granules, prills and AN powder forms using SEM, revealing correlations between surface deformations and AN grade [14,16]. Additionally, investigations examined AN morphology, prill caking, rheology and microstructure alterations, highlighting the impact of AN provenance on its properties [7]. Rao et al. divided porous ammonium nitrate(V) into regular, irregular and dense prills based on SEM examination [7]. Onederra and Araos indicated that AN morphology has an impact on the fumes’ composition, which is the result of energetic materials’ decomposition reaction [19]. Landucci et al. concluded that AN utilized in the mining sector, characterized by a 20% void fraction, resulted in enhanced energy efficiency in energetic materials [20]. Moreover, Miyake et al. stated that alterations in the pore diameter influenced the velocity of energetic materials [17], while Fabin and Jarosz confirmed that the morphology and grain size distribution had a similar impact on the energy of energetic materials [21]. Furthermore, Viktorov et al. documented that changes in AN granule microstructure could augment retention capacity [18]. Kwok and Jones determined the impact of AN morphology on absorption by evaluating octane thermodesorption from the AN surface [22]. It was shown by Zawadzka-Małota and Maranda that fertilizer-grade ammonium nitrate was characterized by lower absorption capacities in comparison with prill ammonium nitrate(V) [23].

Collectively, these studies underscore the multifaceted nature of AN properties, impacted by AN form and provenance, with implications across diverse industrial domains. 

This work aims to present the evaluation of AN morphology, including cross-sectioned AN tomography scans dependent on AN provenance. The evaluation result will explain and help in deciding on a proper ammonium nitrate(V) application. Moreover, the results obtained via SEM and tomography combined with the references provided will explain how porosity and surface area may influence the properties of energetic materials applied in the mining industry. 

## 2. Materials and Methods

### 2.1. Materials

Fertilizer-grade ammonium nitrate(V), Sample 1, was supplied by Anvil S.A., (Włocławek, Poland). It consisted of 34.0% nitrogen, equally divided between nitrate and ammoniacal nitrogen forms. Furthermore, it contained 0.2% magnesium as magnesium nitrate. The prill size ranged from 1 mm to 3 mm, with a bulk density of 920–1000 kg·m^−3^. The absorption index was below 6.0%. Fertilizer-grade AN came in the form of granules. 

Prill ammonium nitrate(V) size 8, Sample 2, was manufactured by Yara International ASA (Szczecin, Poland). It was characterized by 99.5% purity. The remaining 0.5% consisted of coating agents. Moreover, it contained approximately 35.0% nitrogen. The average prill diameter was 1 mm, and the bulk density was 820 kg∙m^−3^. The water content did not exceed 0.3%. The absorption index was in the range of 8–12%.

Prill ammonium nitrate(V) size 7, Sample 3, was also provided by Yara International ASA (Szczecin, Poland). It was also characterized by 99.5% purity and approximately 35.0% nitrogen content. The average prill diameter and bulk density were 0.8 mm and 740 kg∙m^−3^, respectively. The water content did not exceed 0.3%. The absorption index was in the range of 10–14%.

Prill ammonium nitrate(V), Sample 4, was delivered by one of the AN manufacturers. It was characterized by 98.0% purity. The remaining 2.0% consisted of coating agents and organic additives (0.15%). The AN sample contained approximately 35.0% nitrogen. The average prill diameter and the average bulk density were 1 mm and 800 kg∙m^−3^, respectively. The water content did not exceed 0.5%. The absorption index was ca. 10.0%.

All prill AN samples came in the form of prills. 

### 2.2. Methods

X-ray powder diffraction (XRD) patterns were obtained using a PANalytical X’Pert PRO MPD (Malvern Panalytical Ltd., Malvern, UK) system. CuKα radiation was applied at 40 kV and 30 mA. XRD scanning was performed in the 5–50° 2θ range with a 0.033° step size. All ammonium nitrate(V) samples came in powder form and were placed in appropriate holders. XRD measurements were conducted at room temperature.

Scanning electron microscopy (SEM) micrographs were obtained using a Nova NanoSEM 200 (FEI Company, Hillsboro, OR, USA) operating at 5–18 keV. Prior to SEM analysis, all samples were coated with a carbon layer. The samples were mounted on holders with double-sided, conductive, vacuum-compatible carbon tape. Images were captured under low vacuum conditions (approximately 60 Pa) with an electron beam voltage of 10 kV. The low vacuum detector (LVC) was utilized in the secondary electron mode for image acquisition.

Tomographic scan was conducted using a GE Phoenix v|tome|x M device (General Electric Company, Hürth, Germany), utilizing X-ray spectroscopy technology. The samples were secured on low-absorption foam via adhesive contact, which was then positioned on a sample holder and fixed on the movable stage inside the tomography chamber. The tomography process involves placing the sample between the radiation emitter (tube) and the panel detector. According to projection principles, placing the sample closer to the tube increases magnification, thereby enhancing data quality. The chosen configuration enabled scans with a voxel size of 4.23 µm^3^. Subsequently, radiation power and detector parameters were set uniformly across all scans. The radiation was generated from a microfocus tube operating at 40 kV and 120 mA. During the examination, the sample rotated 360° around the vertical axis over approximately 60 min, resulting in 2600 X-ray projections.

The next phase involved reconstructing the three-dimensional volume from the obtained 2D projections. During a full rotation, the device produced numerous high grayscale images (14-bit for the GE Phoenix v|tome|x M). Standard reconstruction protocols included advanced algorithms, such as beam hardening correction, automatic geometry calibration and geometry optimization [24]. Data reconstruction was performed using Phoenix datos|x 2 rec software (version 2.7.2 RTM (2019)), applying the geometry calibration algorithm, beam hardening correction with a power of 6.3 (BHC+) and automatic geometry calibration (AGC). Further analysis was executed within the VGStudio MAX 3.5.1 environment.

The initial step in processing the reconstructed data involved determining the isovalue corresponding to the material boundary. This value was individually assessed for each sample based on the histogram. The threshold value for the material allowed for direct quantification of the material volume and surface area within the entire sample volume, facilitating subsequent analyses. Porosity was identified as a critical parameter. In the 3D data, porosity was determined using image analysis algorithms, specifically the VGDefX/Only procedure, with a deviation factor set to −0.75. Significant pores were defined as those with a volume exceeding 4 voxels.

The VGDefX analysis simultaneously considered both closed and open porosity, detecting pores extending to the external surface. This analysis is commonly used, and its description is provided in [25,26,27].

**Figure 1:**
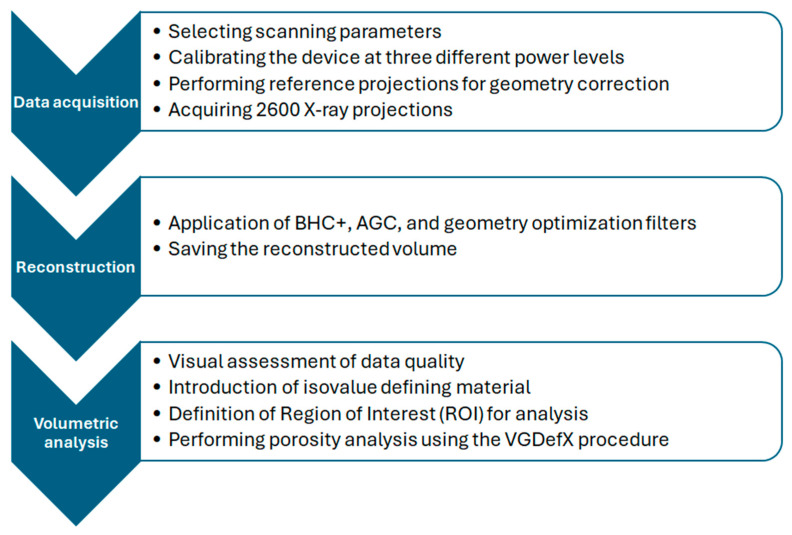
Flowchart of tomography analysis.

The flowchart of tomography measurements is presented in Figure 1. 

## 3. Results and Discussion

### 3.1. XRD Analysis

**Figure 2:**
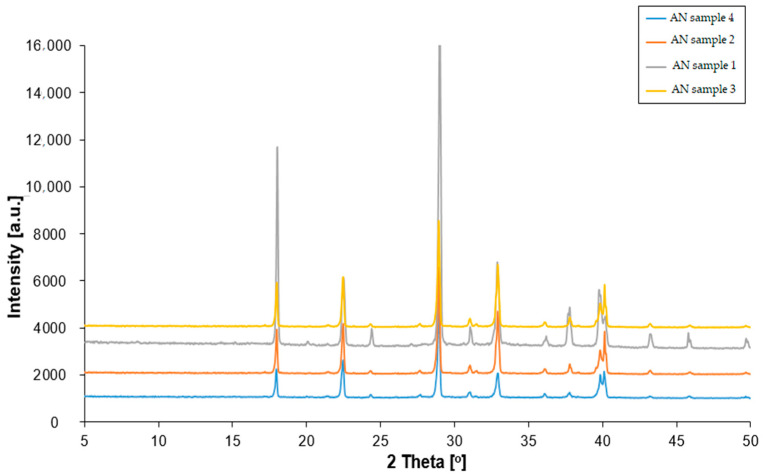
XRD patterns of fertilizer and prill ammonium nitrate(V).

The X-ray diffraction analysis results revealed that ammonium nitrate(V) crystalized within an orthorhombic system, exhibiting P_mmm_ symmetry with two particles per unit cell and belonging to the C_2v_ symmetry class for both NH_4_^+^ and NO_3_^−^ ions (Figure 2). For all ammonium nitrate samples, the diffraction patterns exhibited reflections at specific 2θ angle values, namely 18°, 22°, 24°, 29°, 31°, 33°, 36°, 38°, 40°, 43° and 47°. These reflections corresponded to lattice planes indexed as (100), (011), (110), (111), (002), (020), (102), (201), (112), (211) and (210), respectively. Notably, the diffraction patterns of pure ammonium nitrate remained consistent, irrespective of its provenance. The only difference between prill and fertilizer ammonium nitrate(V) was related to the peaks’ intensity. Fertilizer-type AN was characterized by a chemical composition different to the prill analog of AN. Hence, the presence of magnesium and calcium might overlap and amplify the peak values.

### 3.2. Results of SEM

**Figure 3:**
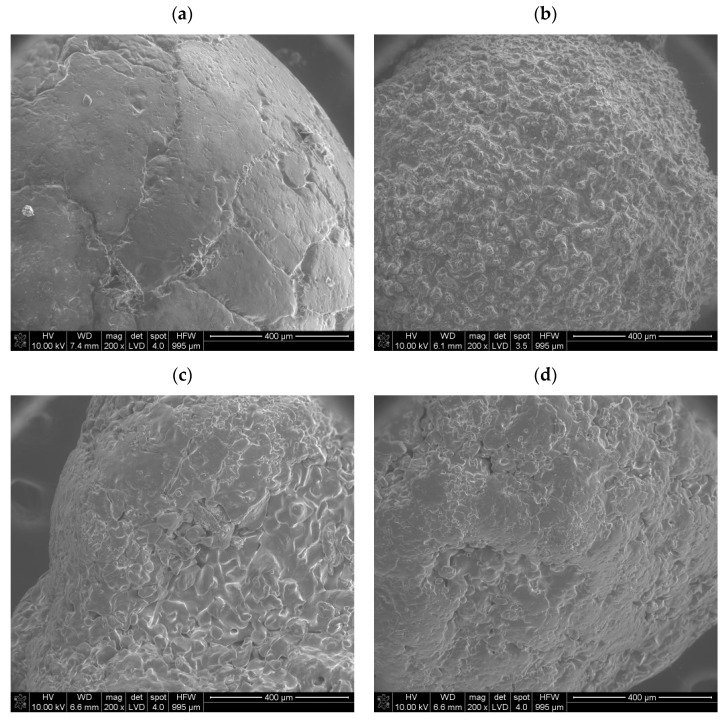
SEM images of various ammonium nitrate(V) prills and granules: (**a**) AN Sample 1, (**b**) AN Sample 2, (**c**) AN Sample 3, (**d**) AN Sample 4.

The SEM analysis showed that the ammonium nitrate(V) samples researched generally had surface deformations and small air voids on the crystal surface; however, their number and character differed in terms of ammonium nitrate(V) provenances. Ammonium nitrate(V) commonly used as fertilizer (Figure 3a) indicated that the granule surface was rather plain, with few surface deformations, e.g., cracks. The observed cracks sometimes crossed themselves and formed larger cavities and forms of wrinkled structures. This corresponds with Rao et al.’s dense prill type [7] and Lotspeich and Petr’s [14] findings. Moreover, Rao et al. indicated that granules delineated by irregular shapes with a smooth crystal surface are more prone to caking [7]. Furthermore, Lotspeich and Petr, as well as Biessikirski et al., observed that cross-sectioned fertilizer-type ammonium nitrate(V) exhibited a longitudinal orientation of crystals, which displayed a complex and rather flat structure. They concluded that some surface deformations resembling wrinkled structures were also evident on the cross-sectioned crystal surfaces [14,28]. Notably, the spaces observed between the crystals resembled channels, indicating a pumice stone. Interestingly, none of these channels was visible on the crystal surface, which corresponds with Figure 3a. This suggests either a possible surface solidification process of the granule or, what is more likely, the presence of a coating agent on the granule crystal surface [29]. By comparing Figure 3a with an image of a coated granule of fertilizer-grade ammonium nitrate(V) in Ref. [23], the presence of a coating agent on the AN’s crystal surface can be deduced. Moreover, based on the SEM result, it can be preliminarily concluded that this type of ammonium nitrate(V) should be characterized by a low absorption index. Contrary to the fertilizer-type ammonium nitrate(V), the porous prill ammonium nitrate(V) (Figure 3b–d) indicated a complex crystal surface. On the prill surface, a large number of surface deformations were visible. The structure, independent of the provenance of the prill sample, was characterized by wrinkles and a number of cracks that crossed each other and merged. The structured surface corresponds with the deformation observed in [14,23,30]. Visible crystals on each prill surface are characterized by irregular and angular shapes. According to Rao et al., this may lead to formation of strong bonds and increased caking [7]. The only difference between ammonium nitrate(V) Sample 2 and Sample 3 was that, in the case of a smaller prill diameter, the number of deformations for a similar crystal surface was greater in contrast to the larger diameter grain. This should extend the potential surface contact and absorption capacities. In comparison with the ammonium nitrate(V) Sample 4, no differences were visible. A similar structure and lack of a central cavity were observed. Moreover, Lotspeich and Petr, as well as Biessikirski et al., indicated that cross-sectioned prill ammonium nitrate(V) also had intricate features, including wrinkled structures and air gaps between crystals. Notably, these air gaps were longitudinally aligned with the prill core and small deformations, such as cracks, on the surface of the cross-sectioned crystals. Lotspeich and Petr’s research work indicated the presence of steam-like pathways traversing the prill body. These pathways were a result of the extended residence time of ammonium nitrate(V) drops during descent down a tall tower [14,16].

Viktorov et al. indicated both the provenance and the influence of thermal treatment on porization, which in the end resulted in a change in absorption. They made an evaluation of fertilizer-grade ammonium nitrate(V) and porous prill AN before and after thermal treatment. At first, Viktorov et al. indicated that the index label GOST No. 2-2013 ammonium nitrate(V) exhibited a smooth or glassy surface, representing an irregular fusion of ammonium nitrate aggregates. They observed that the junctions between these fused aggregates were intricate, forming irregularly shaped polygons ranging in size from 50 to 300 µm. The cracks, occurring at distances of 200–300 µm, were randomly distributed and often partially coalesced. The inner surface of the shrinkage cavity shared similarities with the granule surface [18]. The thermal treatment, or porization, was a structural transformation that occurred both in the surface and body of the granules. Granules labeled as PorAN demonstrated surface cracks spanning up to 100–150 µm in width, dividing the surface into blocks sized between 100 and 500 µm. Victorov et al. concluded that these blocks were vertically displaced relative to each other, resulting in a loss of glossiness and a decrease in bulk density. Larger cracks tended to close, while the mouth of the shrinkage cavity developed a network of smaller cracks, measuring 15–20 µm in width. The internal structure was displayed through cracks connecting the surface to the shrinkage cavity [18]. Two samples labeled by Victorov et al. as PAN were produced, respectively, using specialized manufacturing techniques, such as PAN-MH and PAN-GP. Victorov et al. interpreted ammonium nitrate(V) as a granular structure, characterized by a fusion of crystal blocks sized between 200 and 300 µm. The granule surface looked heterogeneous and lumpy, with composite interfaces between blocks forming randomly arranged bodies. Similarly, the surface around the shrinkage cavity resembled that of the granule surface. These granules might exhibit junctions of aggregates with porous structures, and the internal structure reveals numerous rounded interfaces containing void inclusions [18]. The complex structure in either case showed the possible higher absorption capacities in contrast with the fertilizer ammonium nitrate(V). 

### 3.3. Results of Tomography

**Figure 4:**
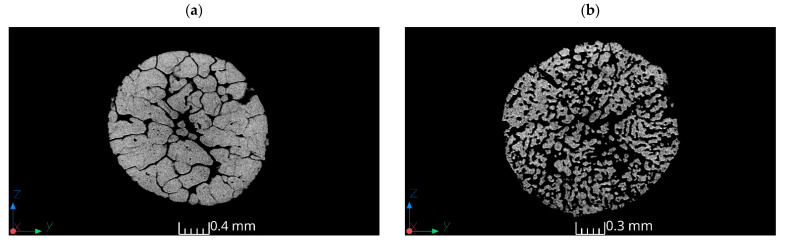

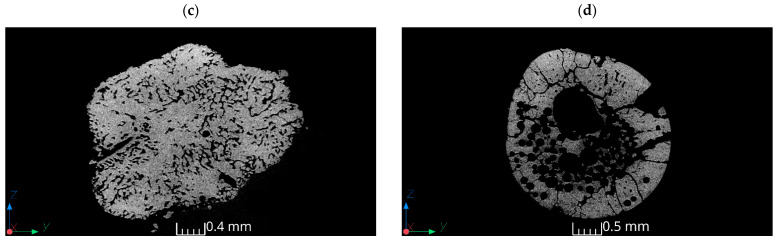
Tomography XY cross-sectioned data of various ammonium nitrate(V) prills and granules: (**a**) AN Sample 1, (**b**) AN Sample 2, (**c**) AN Sample 3, (**d**) AN Sample 4.

The tomography results showed that the structures of samples exhibited significant differences. Fertilizer ammonium nitrate(V) (Figure 4a) was characterized by open porosity, with interconnected channels permeating the particle. These channels did not follow a straight path and had diameters locally exceeding 250 µm. Additionally, closed pores with diameters ranging from 9 µm to 70 µm were present. This confirms Leonard et al.’s results, where the pore diameter of fertilizer-grade AN was in the range of 1–500 µm. However, Leonard et al. observed few inclusions and few pores throughout the prill [3]. The size of the pores has a direct impact on surface contact, which affects absorption capacity. The surface of Sample 1, both internal and external, lacked high roughness features. This is in line with the SEM results of fertilizer-grade ammonium nitrate(V) (Figure 4a). Cross-sectional images suggested that the voids resulted from cracks formed during the production process rather than from inherent porosity. Moreover, the presence of small rounded voids confirms the pumice structure observed in the cross-section images by Lotspeich and Petr [14], as well as by Pereze-Garcia et al. [4]. 

The tomography results revealed that prill ammonium nitrate(V) samples (Figure 4b–d) exhibited a somewhat similar morphology; however, some differences between the samples were visible. Ammonium nitrate(V) Sample 2 contained numerous pores with diameters ranging from 20 µm to 150 µm. The pores were uniformly distributed and interconnected, creating relatively large void spaces within the sample. This uniform distribution of pores resulted in a high tendency toward open porosity, leading to high roughness on both internal and external surfaces due to the prevalence of spherical voids throughout the volume. Ammonium nitrate(V) Sample 3 exhibited an irregular shape with a varied pore arrangement. The pores were characterized by low sphericity. A cross-sectional analysis revealed local elongation in one direction, although no dominant elongation direction was evident throughout the sample. The surface of Sample 3 featured depressions that contributed to increased open porosity. In the case of both samples (AN Sample 3 and Sample 4), the pore network exhibited more rounded edges. This corresponds with the observation presented by Leonard et al. concerning ammonium nitrate(V), which is applied in the mining industry [3].

Sample 4 was distinguished by the highest amount of voids, featuring channels similar to those observed in Sample 1, as well as both spherical and elongated pores. The largest pore had a diameter of 0.7 mm. The amalgamation of all existing pores led to the merging of void volumes, resulting in high open porosity. Similar to Leonard et al.’s results [3], the existence of a central cavity in the upper part of the prill (Figure 4d) was noticed. The presence of a central cavity was not observed in AN Samples 1–3. However, contrary to Leonard et al.’s findings, small spherical voids, which were probably formed during the prilling process, were noticed in AN Sample 4. These small voids can probably merge while subjected to elevated temperature and may probably form one large central cavity. This would correspond with a cavity in the PAN-GP sample [18], as well as with the AN sample examined by Zawadzka-Małota and Maranda [23]. An extensive inert surface of the prill confirmed the pumice and funnel structure that was reported in [14,28]. This extended surface area of prill ammonium nitrate(V) would result in greater absorption capacities in comparison with fertilizer-grade ammonium nitrate(V).

The extended porosity results of the tomography analysis are presented in Table 1.

The results of the conducted analyses indicated that prill ammonium nitrate(V) Sample 4 exhibited the highest porosity, exceeding 70%, while prill AN Sample 3 demonstrated the lowest porosity at 13.45%. The material surface characteristics depended not only on porosity but also on particle shape, as evidenced by Sample 3, where its irregular shape resulted in a significant increase in the surface area. Increased porosity in the cross-sectioned area would result in higher absorption capacities. Due to the varied porosity and particle size of the scanned particles, additional information on the surface-to-volume ratio of the material was included [31]. The highest surface-to-volume ratio was determined for Sample 2. Successively lower values of the surface-to-volume ratio were obtained for Sample 3, Sample 4 and Sample 1. Notably, the ratio obtained for Sample 2 was more than three times larger than the smallest value obtained for Sample 1. The obtained results are in line with Zawadzka-Małota and Maranda’s research, which concluded that fertilizer ammonium nitrate(V) had the lowest total pore volume in comparison with the prill AN. They concluded that when the presence of voids in the tested samples increased, their density decreased, leading to an enhanced capacity for oil absorption [23]. In other words, this confirmed that the fertilizer ammonium nitrate(V) would be characterized as a low-absorption type, contrary to prill AN, which would result in its application. Furthermore, by analyzing the porosity ratio, it can be stated that porosity has a direct impact on the possible application of AN. Miyake et al. stated that a decrease in particle diameter results in an increase in the specific surface area, which leads to an increase in the decomposition reaction velocity [17]. Moreover, they concluded that the velocity of the decomposition reaction increases when the mode pore diameter decreases [17]. Based on this, it can be stated that, taking into consideration the extended surface area and open porosity of all porous prill AN samples, AN Samples 3 and 4 would probably have the highest decomposition reaction velocity if applied as oxidizing components in a highly energetic material. 

## 4. Conclusions

The conducted research indicated that the provenance of ammonium nitrate(V) influenced its morphology and resulted in varying absorption capacities. Depending on its absorption capacity, ammonium nitrate(V) can be applied either as a fertilizer (lower absorption AN) or as a component in non-ideal energetic materials (higher absorption AN).

X-ray diffraction (XRD) analyses showed that the manufacturing process and provenance did not affect the crystallite structure. All tested samples exhibited P_mmm_ symmetry with reflections at consistent 2θ angle values.

The scanning electron microscopy (SEM) analysis revealed that prill ammonium nitrate(V) had a complex structure. The crystal surfaces displayed folds with visible cracks and pores. In contrast, the surface of fertilizer-grade ammonium nitrate(V) granules was relatively smooth, with fewer deformations. Victorov’s research indicated that thermal treatment could increase the potential zone of chemical reactions by extending the deformations.

The tomography analysis showed that fertilizer-grade ammonium nitrate(V) was characterized by open porosity, with interconnected channels permeating the granule. The surface-to-volume ratio was the lowest (21.17 mm^−1^), which, with a rather smooth crystal surface, limited the surface area and indicated that fertilizer-grade ammonium nitrate(V) should have the lowest absorption ratio (disclosed as up to 6% by the producer). Prill ammonium nitrate(V) samples varied in the cross-section. In the case of the same prill size and different prill manufacturers, the impact of the manufacturing process was evident. The highest number of voids was found in the porous prill AN Sample 4, including both spherical and elongated pores. The amalgamation of these pores resulted in high open porosity. In contrast, in ammonium nitrate(V) manufactured by Yara International ASA, the existing pores did not merge as extensively as in a similar sample manufactured by another supplier (Sample 4). This can be a result of maintaining the prill at an elevated temperature for longer, which increases the surface area. Longer prilling would probably extend the central cavity observed in the porous prill Sample 4.

The largest surface area was observed in smaller diameter prill size AN (358 mm^2^) and in porous ammonium nitrate Sample 4 (329 mm^2^). A larger surface area combined with observed open porosity indicates that these prills should have the highest absorption capacity; however, this should be proven by the S_BET_ measurement. The obtained results confirmed the information provided by manufacturers concerning the absorption ratio (up to 14%), as well as the observations provided by Miyake et al. [17]. 

Both SEM and tomography results indicate that fertilizer-grade ammonium nitrate(V) has a rather flat surface, with a low number of deformations. The tomography images revealed channels that meander throughout the granule and which should connect with granule surface. However, no voids were visible under SEM. This indicates the presence of a coating agent. Contrary to fertilizer-grade AN, all porous prill samples confirmed the extended surface both in the cross-section and on the prill surface, which influences absorption capacity.

Future work should additionally aim to investigate the specific surface area (S_BET_) of both types of ammonium nitrate(V). This measurement will indicate a direct impact of sample morphology on the absorption capacity. 

## Figures and Tables

**Table 1:** Results of the tomography analysis.

Sample	Volume of the Material (mm^3^)	Surface Area of the Material (mm^2^)	Porosity (%)	Mean Diameter (mm)	Surface-to-Volume Ratio (mm⁻¹)
1	5.48	115.99	48.87%	2	21.17
2	2.1	144.74	62.93%	1.6	68.92
3	8.65	358.05	13.45%	3	41.39
4	9.1	329.6	70.63%	3.2	36.22

## Data Availability

The original contributions presented in the study are included in the article, further inquiries can be directed to the corresponding author.
